# A Breath of Relief: Oxymetazoline and Flunisolide Nasal Spray in the Management of Myasthenia Gravis Ptosis

**DOI:** 10.7759/cureus.58812

**Published:** 2024-04-23

**Authors:** Toni Aloyan, Najla Shilleh, Arjun Sharma, Barsam Barsamian, Lisa Sovory

**Affiliations:** 1 Neurology, Arrowhead Regional Medical Center, Colton, USA; 2 School of Medicine, California University of Science and Medicine, Colton, USA; 3 Internal Medicine, Arrowhead Regional Medical Center, Colton, USA

**Keywords:** treatment options, treatment, flunisolide, oxymetazoline, myasthenia gravis (mg), eye ptosis

## Abstract

The current pharmaceutical management of myasthenia gravis (MG) is widely accepted to be pyridostigmine and prednisone, both known to cause adverse effects and incur significant costs. This treatment may be particularly burdensome for patients primarily complaining of localized ocular MG, and little is known about the management of MG ptosis with topical medications. Oxymetazoline hydrochloride 0.1% ophthalmic solution has recently been approved by the FDA for the treatment of ptosis, but there have been limited studies in MG ptosis and no report to date of symptomatic improvement with the intranasal formulation. This case report discusses a 71-year-old female whose newly diagnosed MG ptosis resolved after three days of intranasal oxymetazoline hydrochloride 0.05%, followed by three days of intranasal flunisolide. Our patient’s rapid resolution of symptoms, along with the favorable side effect profile and over-the-counter availability, highlights the promising indication for the use of intranasal oxymetazoline and flunisolide as potential alternatives or adjuncts in MG management. Further research in larger cohorts is necessary to confirm the efficacy of these nasal sprays in treating MG ptosis.

## Introduction

Despite the prevalence of ocular myasthenia gravis (MG), literature on the acute treatment of these symptoms is sparse [1-3]. MG is an autoimmune disorder characterized by autoantibodies against post-synaptic acetylcholine receptors at neuromuscular junctions (NMJ) [4-6]. Ptosis is a classic symptom of MG and a significant cause of morbidity in patients [2-4]. In MG patients, ptosis is characterized by downward displacement of the upper eyelids that progressively worsens throughout the day [2,6]. Ptosis is the initial presenting symptom in over half of patients with MG and is eventually present in over 90% of patients with the condition [6]. This symptom is associated with a significant negative psychosocial impact in patients with MG [7,8]. One study that examined survey data from 166 MG patients found that ptosis made them feel stigmatized in public and increased their social anxiety. In addition, a common theme among MG patients was a desire for healthcare professionals to provide more direct support for their ptosis [8].

The current standard of care for patients with MG includes acetylcholine esterase inhibitors, such as pyridostigmine, and glucocorticoids for severe cases [9]. Randomized clinical control trials and extensive retrospective data have shown efficacy in improving ocular symptoms for these patients. However, these medications are associated with significant side effects. Therefore, a safe and easily accessible option is needed to help these patients manage localized symptoms, such as ptosis.

Oxymetazoline is a direct-acting alpha-adrenergic agonist with affinity to both α1- and α2-receptors [10]. With its nonselective sympathomimetic and vasoconstrictive properties, the intranasal spray is available over the counter as a nasal decongestant and can be used by otolaryngologists as a topical hemostatic agent [11]. In July 2020, the ophthalmic solution of oxymetazoline (Upneeq) became the first FDA-approved medication for blepharoptosis [12]. Flunisolide (Nasalide) is an over-the-counter nasal corticosteroid with anti-inflammatory properties commonly used to treat allergic rhinitis [13]. To our knowledge, this is a rare study to explore the use of nasal oxymetazoline and flunisolide in managing MG-associated ptosis. In this case report, we add to the literature by presenting a case of newly diagnosed MG with ptosis that responded to a sequential approach of oxymetazoline followed by flunisolide nasal spray.

## Case presentation

A 71-year-old female patient with a past medical history of hypertension, diabetes mellitus, and Takotsubo cardiomyopathy was admitted to the emergency department, presenting with a three-week history of left eye ptosis accompanied by progressive dysphagia over the preceding two weeks. The patient exhibited associated symptoms, including diminished strength in the tongue and jaw muscles, leading to an impaired ability to swallow both solids and liquids efficiently. This condition exacerbated phlegm accumulation, triggering episodes of dyspnea and choking. Further complaints included an inability to expectorate, constipation two days before presentation, and significant weight loss of 23 pounds over four weeks. Her physical exam was significant for a positive ice pack test in her left eye. The patient had seen an ophthalmologist before admission who had ruled out ophthalmologic causes for her ptosis. In addition, it was important to consider intracranial and infectious pathologies, such as cranial nerve compression or complications from sinus infections. Given the broad differential for dysphagia, including brain stem stroke and tumor in addition to MG, a brain MRI, esophageal endoscopy, and laryngoscopy were done to rule out other etiologies. To confirm our diagnosis, an MG and Lambert-Eaton panel, along with a chest CT, were ordered. All imaging and procedural studies were unremarkable with autoimmune panels pending.

The patient’s home medications to manage her chronic conditions remained unchanged and included aspirin, irbesartan, metoprolol, atorvastatin, and furosemide. For symptomatic treatment of her dysphagia, congestion, and increased phlegm production, the patient was initially treated with oxymetazoline (Afrin) 0.05%, starting with three sprays per nostril on the first day, then two sprays per nostril twice daily, and was discontinued on the third day to preempt rebound congestion. Due to the patient’s inability to tolerate oral intake, nasogastric (NG) tube insertion was necessitated, resulting in improved well-being following nutritional supplementation. Subsequently, the patient was administered two sprays of flunisolide (Nasalide) per nostril twice daily from the third to the ninth day to alleviate nasal congestion.

**Figure 1:**
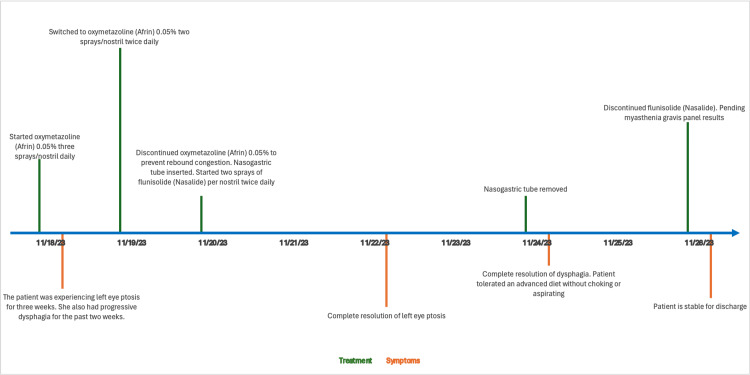
Treatment algorithm and symptom resolution timeline

Beginning on the fifth day, the left eye ptosis completely resolved. The NG tube was removed on the seventh day, following which the patient managed an advanced diet without choking or aspiration. There was a complete resolution of dysphagia by the seventh day (Figure 1). Remarkably, symptom alleviation was achieved without the administration of pyridostigmine. The patient was discharged pending the results of a myasthenia panel, which ultimately confirmed a diagnosis of MG, evidenced by positive acetylcholine receptor binding antibodies from the MG panel.

## Discussion

This report presents the first documented improvement in ptosis associated with MG following the use of intranasal oxymetazoline and flunisolide spray. Individuals with MG often undergo prolonged treatment with immunosuppressive therapy and/or pyridostigmine, both of which are associated with significant adverse effects. As ocular symptoms are characteristic of MG, emerging data on the use of oxymetazoline 0.1% eye drops indicate its potential effectiveness in treating ptosis secondary to MG.

A pooled analysis of two randomized, double-blind clinical trials with 304 patients demonstrated rapid upper eyelid elevation with once-a-day oxymetazoline 0.1% eye drops and sustained elevation for 42 days in patients with acquired ptosis [8,14]. The results showed statistically significant differences in the marginal reflex distance 1 (MRD1) at 5- and 15-minutes post-application on days 1, 14, and 42 and at two- and six-hour post-application on days 1 and 14. Additionally, fewer adverse events were in the treatment group versus placebo (31 vs 35.6%), and 81% were considered mild [15]. Although patients with ptosis secondary to MG were excluded from this study, there have been case reports to date that have shown the efficacy of oxymetazoline 0.1% eye drops in MG ptosis, specifically in patients whose symptoms were refractory to pyridostigmine and prednisone [1,2]. To the best of our knowledge, our case report stands out as the first to explore the potential use of oxymetazoline intranasal formulation for the symptomatic treatment of MG ptosis.

In addition to oxymetazoline, our patient used nasal flunisolide, which has a more clearly defined impact on the disease course of MG. Intranasal steroids have been shown to improve ocular symptoms associated with allergic rhinitis without a clear mechanism of action [16]. Another notable case report discusses the complete resolution of a patient’s ptosis due to isolated cranial nerve palsy secondary to acute sinusitis after one week of intranasal steroids, oxymetazoline, and nasal douching [17]. Although oxymetazoline 0.1% eye drops are known to improve ptosis by activating alpha-adrenergic receptors in the Muller’s muscle that elevates the upper eyelid, the mechanism by which intranasal sprays provide relief of ocular symptoms remains unclear [2]. Although significant absorption through the nasolacrimal system is unlikely due to gravity, the upward force exerted by spray bottles and some retrograde flow via nasolacrimal cilia may make it plausible. Another possibility lies in the therapeutic benefits of systemic absorption, likely due to vasoconstrictive effects improving muscle tone. It is crucial to acknowledge that there have been only transient side effects of flunisolide such as nasal stinging and throat irritation [18]. Considering the systemic absorption of lipophilic drugs like oxymetazoline, potential adverse effects include increased nasal congestion, impaired vision, fast or irregular heartbeat, hypertension, and dizziness [19]. Furthermore, side effects such as reversible segmental cerebral vasoconstriction and severe headaches have been reported, especially in adults. Fortunately, our patient did not report any adverse effects.

The rapid resolution observed in our case, along with the favorable side effect profile and over-the-counter availability of oxymetazoline and flunisolide, underscores their potential as safe and cost-effective options for managing MG-related ptosis. This case offers a new perspective on the symptomatic treatment of MG, potentially advancing the standard of care and thereby enhancing the quality of life for these patients. Using two intranasal sprays sequentially introduces a limitation to our findings as it is unclear which spray contributed more significantly to the favorable outcome. The findings in this report highlight the need for further research through randomized clinical control trials to elucidate the clinical significance of these treatments more clearly.

## Conclusions

This case report showcases a promising indication for the use of intranasal oxymetazoline hydrochloride 0.05% and intranasal flunisolide as a symptomatic treatment for ptosis in patients with MG. Our patient with MG showed complete resolution of her ptosis with just three days of intranasal oxymetazoline followed by three days of intranasal flunisolide. This case is particularly noteworthy due to the rapid relief and ease of application, suggesting these treatments as potential alternatives or adjuncts in MG management. Further studies with larger patient populations and controlled settings are necessary to clarify the use of oxymetazoline and flunisolide intranasal sprays for ptosis, specifically in relation to MG, and to explore their mechanism of action, long-term efficacy, and safety profile.
